# Quantitative Structure − Antiprotozoal Activity Relationships of Sesquiterpene Lactones ^†^

**DOI:** 10.3390/molecules14062062

**Published:** 2009-06-08

**Authors:** Thomas J. Schmidt, Amal M. M. Nour, Sami A. Khalid, Marcel Kaiser, Reto Brun

**Affiliations:** 1 Westfälische Wilhelms-Universität Münster, Institut für Pharmazeutische Biologie und Phytochemie, Hittorfstraße 56, D-48149 Münster, Germany; E-mails: amal_mukhtar@hotmail.com (A-M.N.); 2 University of Khartoum, Department of Pharmacognosy, Faculty of Pharmacy, P.O. box 1996, Khartoum, Sudan; E-mail: khalidseek@hotmail.com (S.K.); 3 Swiss Tropical Institute (STI), Socinstrasse 57, CH-4002 Basel, Switzerland;E-mails: Marcel.Kaiser@unibas.ch (M.K.), Reto.Brun@unibas.ch (R.B.)

**Keywords:** *Trypanosoma*, *Leishmania*, *Plasmodium*, antiprotozoal activity, sesquiterpene lactone, QSAR

## Abstract

Prompted by results of our previous studies where we found high activity of some sesquiterpene lactones (STLs) against *Trypanosoma brucei rhodesiense* (which causes East African sleeping sickness), we have now conducted a structure-(*in-vitro*)-activity study on a set of 40 STLs against *T. brucei rhodesiense*, *T. cruzi*, *Leishmania donovani* and *Plasmodium falciparum*. Furthermore, cytotoxic activity against L6 rat skeletal myoblast cells was assessed. Some of the compounds possess high activity, especially against *T. brucei* (e.g. helenalin and some of its esters with IC_50_-values of 0.05-0.1 µM, which is about 10 times lower than their cytotoxic activity). It was found that all investigated antiprotozoal activities are significantly correlated with cytotoxicity and the major determinants for activity are α,β-unsaturated structural elements, also known to be essential for other biological activities of STLs. It was observed, however, that certain compounds are considerably more toxic against protozoa than against mammalian cells while others are more cytotoxic than active against the protozoa. A comparative QSAR analysis was therefore undertaken, in order to discern the antiparasitic activity of STLs against *T. brucei* and cytotoxicity. Both activities were found to depend to a large extent on the same structural elements and molecular properties. The observed variance in the biological data can be explained in terms of subtle variations in the relative influences of various molecular descriptors.

## 1. Introduction

**Figure 1:**
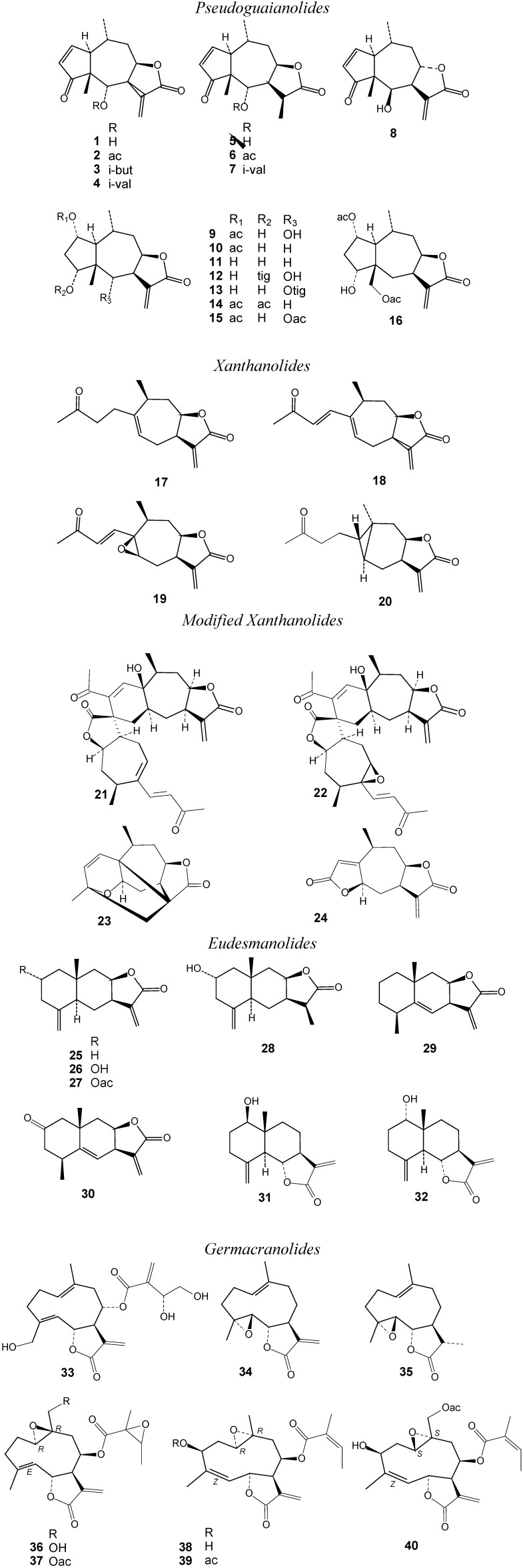
Structures of the sesquiterpene lactones under study.

Protozoal infections such as malaria, trypanosomiases and leishmaniases represent major health risks in developing countries. It is estimated that world-wide these diseases are responsible for over one million deaths a year [1]. While relatively effective and safe therapies for malaria exist, African sleeping sickness and Chagas’ disease caused by *Trypanosoma* species, as well as cutaneous and visceral Leishmaniasis (Kala-Azar) are currently classified as “neglected diseases” [2]. Only a few effective drugs exist for the treatment of these infections and therapy is often accompanied by severe adverse effects and high toxicity, so the search for new drugs or lead structures, especially against *Trypanosoma* and *Leishmania* infections, is an urgent task [3]. Natural products have in many instances been found to provide interesting leads for such diseases [3]. Among many other examples, it has been shown by our group that certain sesquiterpene lactones (STLs) possess considerable activity against *Trypanosoma* species [4,5]. The present study was conducted in order to obtain a more detailed insight into the structure-activity relationships governing antiprotozoal activity of STLs. To this end, 40 STLs including 16 pseudoguaianolides, four xanthanolides, four modified xanthanolides, eight eudesmanolides and eight germacranolides (see Figure 1) were tested *in-vitro* against four major protozoan pathogens, *Trypanosoma brucei rhodesiense* (*Tbr*), *Trypanosoma cruzi* (*Tcr*), *Leishmania donovani* (*Ldon*) as well as *Plasmodium falciparum* (*Pfc*). As a control system to assess cytotoxic activity, the rat skeletal myoblast cell line *L6*, also serving as host cell system in the *Tcr* assay, was used. The resulting data were subsequently investigated for quantitative structure-activity relationships (QSAR) using molecular modelling and multivariate data analysis tools.

## 2. Results and Discussion

### 2.1. Biological Activity Data and Activity-Activity Relationships

The bioactivity data of 40 sesquiterpene lactones (Structures see Figure 1) tested *in-vitro* for activity against *T. brucei rhodesiense* (*Tbr*), *T. cruzi* (*Tcr*), *L. donovani* (*Ldon*) and *P. falciparum* (*Pfc*) as well as cytotoxicity against L6 rat skeletal myoblasts are reported in Table 1. Generally, *T. brucei rhodesiense* was found to be the most sensitive to STLs among the tested parasites. In line with the significant activity of helenalin (**1**), previously reported [4], its ester derivatives **2**-**4** were found to be very active, with IC_50_s in the range of 0.1 µM and below. Pseudoguaianolides of the helenalin series also showed the highest activity against the other parasites. Some of the helenalin congeners exhibited more pronounced bioactivity against *Tcr* than the positive control benznidazole. They also showed activity against *Ldon* and *Pfc* in a similar range as their respective controls.

On the other hand the xanthanolide 8-epixanthatin-1,5-epoxide **19**, recently isolated as the most active compound from *Xanthium brasilicum* in the course of a bioactivity-guided isolation study [5], exhibited considerable activity against *Tbr* and its close relative *Ldon*. However, all of the most active STLs displayed significant toxicity against the rat skeletal myoblast cell line *L6*, used as control to assess cytotoxicity.

**Table 1:** *In vitro* antiprotozoal and cytotoxic activity of the STL under study (IC_50_, µM). Each entry represents the mean of two independent measurements.

Compound	*T. brucei rhodes. (Tbr)*	*T. cruzi* *(Tcr)*	*L. donovani* *(Ldon)*	*P. falciparum* *(Pfc)*	Cytotoxicity *(L6)*
**1**	0.052	0.695	n.t.	n.t.	0.992
**2**	0.063	0.538	0.446	0.329	0.809
**3**	0.105	1.568	0.837	0.700	1.030
**4**	0.116	2.478	0.871	0.822	1.298
**5**	0.686	21.477	3.848	3.047	8.902
**6**	1.415	6.928	>4*	n.t.	3.056
**7**	0.911	3.534	1.476	1.603	4.583
**8**	0.319	1.870	>4*	n.t.	2.443
**9**	9.275	45.833	n.t.	n.t.	12.284
**10**	6.705	20.422	12.240	6.461	4.392
**11**	18.346	52.744	20.602	12.143	31.917
**12**	10.951	49.176	17.967	10.604	16.676
**13**	19.242	60.165	14.505	27.473	31.758
**14**	1.174	14.657	4.257	8.943	8.429
**15**	3.961	16.311	8.675	5.984	7.281
**16**	13.607	44.891	11.831	10.656	21.557
**17**	62.081	>120*	23.831	>20*	178.790
**18**	2.919	26.220	17.642	7.825	30.203
**19**	0.330	11.260	0.599	6.511	22.137
**20**	15.887	38.992	14.597	12.702	19.839
**21**	0.638	16.083	22.047	4.961	9.429
**22**	1.288	95.420	26.908	6.527	13.282
**23**	162.200	>120*	15.400	>20*	>365*
**24**	8.205	>120*	27.051	>20*	427.350
**25**	23.621	22.263	>14*	n.t.	4.483
**26**	7.802	24.778	>4*	n.t.	0.948
**27**	10.724	24.345	11.759	9.207	8.914
**28**	341.480	>120*	>120*	>20*	187.120
**29**	2.625	8.297	3.030	6.401	5.991
**30**	1.602	17.256	8.699	10.691	10.183
**31**	17.806	28.911	6.008	12.056	33.185
**32**	1.107	26.210	7.339	13.629	25.565
**33**	1.303	16.799	15.212	6.323	10.212
**34**	0.388	10.665	3.556	11.895	7.238
**35**	49.000	>120*	35.560	>20*	132.600
**36**	14.283	25.582	2.726	9.444	13.624
**37**	0.478	15.786	5.190	6.333	7.750
**38**	0.942	16.492	18.204	8.812	12.970
**39**	0.804	17.574	30.074	8.069	10.099
**40**	0.698	11.833	17.095	6.643	6.667
*pos. control*	0.008^a^	2.000^b^	0.270^c^	0.248^d^	0.012^e^

*highest concentration tested, IC_50_ not determined. n.t.: not tested. ^a^melarsoprol; ^b^benznidazole; ^c^miltefosine; ^d^chloroquine; ^e^podophyllotoxin.

Pairwise correlation of the bioactivity data (Table 2) revealed that antiprotozoal activity is in all cases significantly correlated with cytotoxicity against the L6 cells. It can thus be expected that all investigated bioactivities are – at least to a significant degree – governed by similar structure-activity relationships as cytotoxicity. However, several of the tested compounds (e.g. **1**-**6**, **18**, **19**) displayed significantly higher levels of antiparasitic activity, especially against *Tbr,* than cytotoxicity, whereas others, e.g. the eudesmanolides **25** and **26**, are considerably more toxic against the mammalian cells than against the protozoa.

**Table 2:** Interrelations of antiprotozoal and cytotoxic activity data (Correlation coefficients R of pIC_50_ data).

	T. b. rhod.	T.cruzi	L. donov.	P. falcip.	L6 cytotox.
**T. b. rhod.**	1.000				
**T.cruzi**	0.851	1.000			
**L. donov.**	0.704	0.823	1.000		
**P. falcip.**	0.808	0.912	0.753	1.000	
**L6 cytotox.**	0.698	0.857	0.697	0.905	1.000

In order to assess the degree of selectivity of each STL against a particular parasite, the ratio of the cytotoxic IC_50_ value of the mammalian control cell line L6 over the respective values for the four protozoan organisms was investigated. The most favourable selectivity indices (SI) with respect to activity against *Tbr* were found in case of compounds **19** and **24**, respectively, which were 67 and 52 times more active than cytotoxic. These two compounds, moreover, were also the most selective against *Ldon* (SI = 36 and 15, respectively). The absolute activity of compound **24** being relatively low, however, an interesting potential as lead compound may be conceived especially for **19**. Helenalin **1**, followed by its acetate **2**, both showing *Tbr* activity well below 0.1 µM, despite their somewhat higher cytotoxicity still possess SI values of 19 and 13, respectively, rendering them also interesting candidates for further studies.

### 2.2. Structure-activity relationships, QSAR

A very simple but nevertheless essential structure-activity relationship (SAR) is already obvious when visually comparing the structures and their activity data. Compounds possessing at least one potentially reactive α,β-unsaturated carbonyl group as a pharmacophore usually show significant antiprotozoal as well as cytotoxic activities, while compounds lacking such structural elements show relatively insignificant activity. The presence of such potential Michael acceptors in the structure is thus a prerequisite for activity, in much the same way as reported in previous studies [6,7,8,9] and in full agreement with the frequent observation that various bioactivities of STLs are associated with their chemical reactivity, especially towards free thiol groups (e.g. cysteine residues in enzymes and transcription factors; for overviews see [8, 9]).

Previous studies on quantitative SAR (QSAR) in our laboratory have concentrated on structure-cytotoxicity relationships among STL and the major structural determinants of this activity of various data sets against several human and murine cell lines were reported [6, 7].

Since the spread of activity data was largest in case of the *Tbr* activity (3.8 log units) and the absolute level of activity displayed by some of the compounds was highest against this parasite, we concentrated on this set of activity data. In our previous work [6, 7] it was found that cytotoxic activity of STLs correlates quite strongly with a very simple type of molecular descriptors, namely, binary indicator variables which encode the presence/absence of a particular reactive structure element. The same was found also in this study. When the activity of all 40 compounds against *Tbr* and their L6-cytotoxicity were analysed for correlation with such descriptors (ML, 1 in case of the presence, 0 in case of absence of an α-methylene-γ-lactone, ENONE for the presence of an α,β-unsaturated ketone structure) by multiple linear regression, squared correlation coefficients (R^2^) of 0.61 and 0.41, respectively, were found. These values being not very high, the significance of both descriptors’ regression coefficients was nevertheless confirmed for both sets of activity data by the results of t- and F-tests. Quite interestingly, when only the subset of structurally closely related compounds **1** – **16** (pseudoguaianolides of the helenanolide series) were considered, the correlation coefficients were much higher (R^2^ = 0.87 and 0.79, respectively for *Tbr* and L6). This result is in line with our previous findings [6, 7] and can be interpreted in a straightforward manner by assuming that in case of compounds possessing very similar molecular structure (in fact the same carbon skeleton and thus similar size, shape and substitution) the modulating influence of other structural factors on bioactivity is small compared to the major impact of chemical reactivity (i.e. presence of enone and methylene lactone groups). Both bioactivities in the set of closely related compounds are dominated largely by these factors, i.e. differences in antitrypanosomal as well as cytotoxic activity can easily be explained by differences in the potential to alkylate biomolecules. However, when STLs of a greater structural diversity (i.e. the whole data set) are considered, the modulating influence of other structural features increases (reflected in the lower degree of direct correlation with the indicators for ML and ENONE). Thus, in order to explain the above-mentioned differences in antiprotozoal and cytotoxic activity for the whole set of compounds, other molecular properties and structural features must be considered.

**Figure 2:**
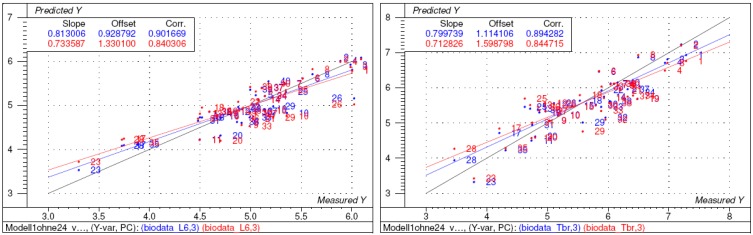
Scatter plots of predicted vs. experimental bioactivity data (pIC_50_; left: *L6* cytotoxicity, right: *Tbr* activity) as obtained by PLS2 after selection of significant variables [model 3 in Table 3]. Blue: calibration data; red: predictions of leave-one-out cross validation.

**Figure 3:**
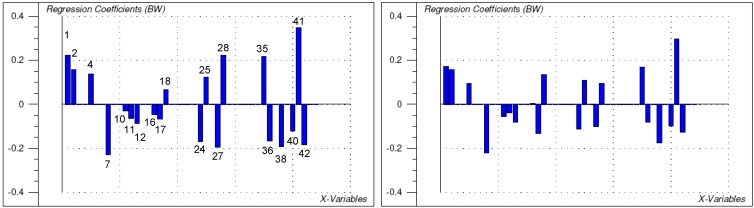
Regression coefficients of the various descriptors (numbering: see Table 4) in the PLS2 model [model 3 in Table 3] (left: *L6* cytotoxicity, right: *Tbr* activity).

**Figure 4:**
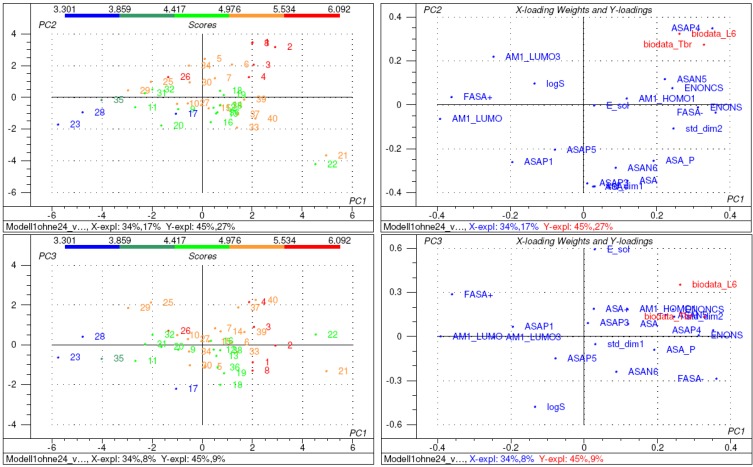
Scores (left) and loading weight plots (right) of the various compounds and the descriptor variables, respectively, in the PLS2 model [model 3 in Table 3]. Top: PC2 vs. PC1, Bottom: PC3 vs PC2. In the scores plots, compounds are coloured according to their activity against Tbr (pIC_50_).

To this end, a 3D model of each molecule was created using the molecular modelling package MOE [11] and a variety of molecular descriptors were calculated using the QSAR module of MOE (for a full list of the descriptors considered see Experimental Section). The resulting data matrix (40 compounds x 44 descriptors) was analysed with the multivariate correlation method PLS2 as implemented in the statistics program The Unscrambler [12]. PLS2 served to correlate both sets of biological activity data simultaneously with the structural descriptors (resulting statistical parameters see Table 3). It was found that compound **24** represented an outlier, leading to poor predictive quality of the correlation model (low cross validated correlation coefficient Q^2^ especially for the cytotoxicity data). After exclusion of this compound and subsequent elimination of variables not significantly contributing to the overall correlation (variable selection by Martens’ uncertainty test [12]), the final model presented in Figure 2, Figure 3 and Figure 4 resulted (statistics see Table 3). In this model, the information content of 20 descriptors is combined in three significant PLS components (PCs, latent variables). The correlation coefficients and leave-one-out cross validated correlation coefficients for Tbr activity are 0.89 and 0.85, respectively, the corresponding values for L6 cytotoxicity are 0.90 and 0.84.

**Table 3:** Statistical parameters of PLS2 models for antitrypanosomal activity (*Tbr*) and cytotoxicity (*L6*).

Model	n var.^a^	n comp.^b^	n PCs^c^	R^2^		Q^2^	
				*Tbr*	*L6*	*Tbr*	*L6*
1	44	40	7	0.83	0.91	0.52	0.41
2	44	39*	4	0.81	0.82	0.66	0.61
3	20	39*	3	0.80	0.81	0.71	0.71

*compound **24** was omitted since it was found to represent an outlier.^ a ^number of variables included; ^b ^number of compounds; ^c ^number of significant PLS components (latent variables). R^2^: squared correlation coefficient; Q^2^: squared correlation coefficient of leave-one-out cross validation.

As can be seen in the loadings plot in Figure 4, the first PLS component (PC1) explaining 45% of the overall variance of the biological data, is dominated by descriptors AM1-LUMO (= energy of the lowest unoccupied molecular orbital; most negative loading) and ENONS (most positive loading). Both descriptors are related to the molecules’ reactivity/alkylating potency. According to frontier molecular orbital theory, electrophilic reactivity is inversely correlated with the energy of a molecule’s lowest unoccupied molecular orbital (LUMO) [13]. Descriptor ENONS, on the other hand represents the molecular surface area due to α,β-unsaturated carbonyl structures which, as expected, has a positive impact on activity.

The second latent variable PC2 (explaining further 27% of the variance in the biological data) receives major influences from descriptors ASAP4 [7] (positive coefficient), ASA, ASA+ and stdim1 (negative coefficients). The former, showing a positive influence also in PC1, represents the molecular surface area attributable to hydrogen atoms attached to the double bond carbons of α,β-unsaturated ketone structures and thus is also related to reactivity and accessibility of the reactive partial structures. The latter three descriptors represent the total surface area, the surface area attributable to atoms with positive partial charge and the largest dimension of each molecule [11]. Accordingly, this PC appears to be related mainly to molecular size. The negative coefficients of the latter descriptors indicate that reduction of the activity is associated with the total size of the molecule coupled with a large overall positive surface area.

Finally, PC3 (explaining further 9% of biological data variance) is clearly correlated with the molecules’ polarity/hydrophilicity. This latent variable receives strong influence from descriptors E_sol (calculated solvation energy, more positive value in case of hydrophobic molecules) and logS (log of the calculated water solubility). The positive coefficient of the former and negative coefficient of the latter descriptors clearly demonstrate an inverse correlation of the biological activities with polarity, i.e. active molecules should not be too polar and but rather have a certain degree of hydrophobicity.

## 3. Conclusions

In conclusion, the very similar PLS coefficients (Figure 3) in the two models for *Tbr* and *L6* activity, which differ only slightly in magnitude for the individual descriptors, clearly show that no major differences exist in the general structure-activity relationships for cytotoxicity and antitrypanosomal activity of the 40 STLs included in the present study. It appears a difficult task –if at all possible - to exploit the relatively subtle structural differences responsible for differential activity with respect to lead structure optimisation. While tests of further STLs and related compounds, as well as the application of further QSAR methods might yet reveal clearer and possibly more detailed quantitative structure-activity relationships, different strategies may have to be applied in order to increase the selectivity and to exploit the interesting antiprotozoal potential of these natural products. In this respect, e.g., the design of parasite-targeted prodrugs [14,15] or exploitation of a parasite transporter may be of interest. Studies in this direction have been initiated.

## 4. Experimental

### 4.1. Test compounds

Compounds **1**–**17**, **20**, **26**-**28** were isolated from *Arnica* species as reported previously [16]. Compounds **18**, **19** and **21**-**24** were isolated from *Xanthium brasilicum* Vell. [5]. Compounds **25**, **29** and **30** were isolated from roots of *Inula helenium* in our laboratory. They were identified by their NMR data which were in accordance with published data [17,18] Compounds **31**-**33** and **36**-**40** originating from various Asteraceae, were kindly provided by G. Willuhn, Düsseldorf, Germany. Compound **35** was kindly provided by N. H. Fischer, Denton, TX, U.S.A. Compound **34** (parthenolide) was obtained from Sigma-Aldrich (cat. No. P667). The purity of all compounds was assessed by ^1^H-NMR, HPLC and/or TLC analyses and found to be >80% in all cases.

### 4.2. In vitro assays and IC_50_ determination

*Plasmodium falciparum*. Antiplasmodial activity was determined using the K1 strain of *P. falciparum* (resistant to chloroquine and pyrimethamine). A modification of the [3H]-hypoxanthine incorporation assay was used [19]. Briefly, infected human red blood cells (final parasitaemia and haematocrit were 0.3% and 1.25%, respectively) in RPMI 1640 medium with 5% Albumax were exposed to serial drug dilutions in microtiter plates. After 48 hours of incubation at 37°C in a reduced oxygen atmosphere, 0.5 mCi 3H-hypoxanthine was added to each well. Cultures were incubated for a further 24 h before they were harvested onto glass-fiber filters and washed with distilled water. The radioactivity was counted using a BetaplateTM liquid scintillation counter (Wallac, Zurich, Switzerland). The results were recorded as counts per minute (CPM) per well at each drug concentration and expressed as percentage of the untreated controls. From the sigmoidal inhibition curves IC50 values were calculated. Assays were run in duplicate and repeated once.

*Trypanosoma brucei rhodesiense and cytotoxicity against L6 cells.* Minimum Essential Medium with Earle’s salts (50 µL) supplemented with 0.2 mM 2-mercapto-ethanol, 1 mM Na-pyruvate and 15% heat-inactivated horse serum was added to each well of a 96-well microtiter plate. Serial drug dilutions were prepared by adding 25 μL complete medium containing 540 μg/mL (6x the starting concentration), thus covering a range from 90 to 0.123 µg/mL. Then 10^4^ bloodstream forms of Trypanosoma brucei rhodesiense STIB 900 in 50 µL of medium were added to each well and the plate incubated at 37°C under a 5% CO_2_ atmosphere for 72 hours. Alamar blue solution (10 µL, 12.5 mg resazurin dissolved in 100 mL distilled water) were then added to each well and incubation continued for a further 2-4 hours. The plate was then read in a Spectramax Gemini XS microplate fluorometer (Molecular Devices Corporation, Sunnyvale, CA, USA) using an excitation wavelength of 536 nm and emission wavelength of 588 nm [20]. Fluorescence development was measured and expressed as percentage of the control. Data were transferred into the graphic programme Softmax Pro (Molecular Devices) which calculated IC_50_ values. Cytotoxicity was assessed using a similar protocol and rat skeletal myoblasts (L6 cells). L6 cells were seeded in to RPMI 1640 medium supplemented with L-glutamine 2 mM, HEPES 5.95 g/L, NaHCO_3_ 2 g/L and 10% fetal bovine serum in 96 well microtiter plates (4,000 cells/well). All following steps were according to the *T. b. rhodesiense* protocol.

*Trypanosoma cruzi*. Rat skeletal myoblasts (L-6 cells) were seeded in 96-well microtiter plates at 2,000 cells/well in 100 µL RPMI 1640 medium with 10% FBS (fetal bovine serum) and 2 mM L-glutamine. After 24 hours the medium was removed and replaced by 100 µL per well containing 5,000 trypomastigote forms of *T. cruzi* Tulahuen strain C2C4 containing the β-galactosidase (Lac Z) gene [21]. Fortyeight hours later the medium was removed from the wells and replaced by 100 mL fresh medium with or without a serial drug dilution. Seven 3-fold dilutions were used covering a range from 90 mg/mL to 0.123 mg/mL. Each drug was tested in duplicate. After 96 hours of incubation the plates were inspected under an inverted microscope to assure growth of the controls and sterility. Then the substrate CPRG/ Nonidet (50 µL) was added to all wells. A colour reaction developed within 2-6 hours and could be read photometrically at 540 nm. Data were transferred into the graphic programme Softmax Pro (Molecular Devices) which calculated IC_50_ values.

*Leishmania donovani* (axenic amastigote assay). Ffty µL of SM medium [22] at pH 5.4 supplemented with 10% heat-inactivated FBS, was added to each well of a 96-well microtiter plate (Costar, USA). Serial drug dilutions in duplicates were prepared covering a range from 30 to 0.041 µg/mL. Then 105 axenically grown *Leishmania donovani* amastigotes (strain MHOM/ET/67/L82) in 50 µL medium were added to each well and the plate incubated at 37°C under a 5% CO_2_ atmosphere for 72 hours. Resazurin solution (10 µL, 12.5 mg resazurin dissolved in 100 mL distilled water) were then added to each well and incubation continued for a further 2-4 hours. The plate was then read in a Spectramax Gemini XS microplate fluorometer (Molecular Devices Cooperation, Sunnyvale, CA, USA) using an excitation wavelength of 536 nm and emission wavelength of 588 nm [20]. Fluorescence development was measured and expressed as percentage of the control. Data were transferred into the graphic programme Softmax Pro (Molecular Devices) which calculated IC_50_ values from the sigmoidal inhibition curves. The compounds used as positive controls in the various bioassays (see Table 1) were of commercial origin, with the exception of melarsoprol, which was a gift from WHO. Their purity (generally > 95%) was specified by the manufacturers.

### 4.3. Computational Methods

3D-models of all compounds were generated with MOE [11] using the MMFF94x force field. A stochastic conformational search was performed for each compound (default settings of MOE) and the resulting conformers were energy minimised using AM1 (MOPAC module of MOE). The conformer with the lowest AM1 energy was used in the QSAR study.

QSAR descriptors were calculated for each of the compounds using the QSAR module of MOE. A full list of descriptors considered is reported in Table 4. For the QSAR analyses, the IC_50_ data for Tbr activity and L6 cytotoxicity, expressed on the molar scale were converted to negative decadic logarithms (pIC_50_).

Multivariate analysis for the resulting data matrix was performed statistics program “The Unscrambler”, v. 9.2 [12] using the PLS2 algorithm which allows simultaneous construction of correlative models for both dependent variables (i.e. pIC_50_ data) using the same set of independent variables (i.e. descriptors). Refinement of the initial model containing all 44 variables was obtained by variable selection based on Martens’ Uncertainty Test as implemented in the Unscrambler program.

**Table 4:** List of molecular descriptors considered in PLS2-QSAR modelling. Descriptors included in the final model [model 3 in Table 3] are highlighted.

**1**	**ENONCS**	accessible surface area of beta carbons in α,β-unsaturated carbonyl structures
**2**	**ENONS**	accessible surface area of α,β-unsaturated carbonyl structures
2	AM1_HOMO	Eigenvalue of highest occupied molecular orbital (MOE/MOPAC/AM1)
**4**	**AM1_HOMO1**	Eigenvalue of second highest occupied molecular orbital (MOE/MOPAC/AM1)
5	AM1_HOMO2	Eigenvalue of third highest occupied molecular orbital (MOE/MOPAC/AM1)
6	AM1_HOMO3	Eigenvalue of fourth highest occupied molecular orbital (MOE/MOPAC/AM1)
**7**	**AM1_LUMO**	Eigenvalue of lowest unoccupied molecular orbital (MOE/MOPAC/AM1)
8	AM1_LUMO1	Eigenvalue of second lowest unoccupied molecular orbital (MOE/MOPAC/AM1)
9	AM1_LUMO2	Eigenvalue of third lowest unoccupied molecular orbital (MOE/MOPAC/AM1)
**10**	**AM1_LUMO3**	Eigenvalue of fourth lowest unoccupied molecular orbital (MOE/MOPAC/AM1)
11	AM1_dipole	Dipole moment calculated with MOE/MOPAC/AM1
**12**	**ASA**	solvent accessible molecular surface area [11]
**13**	**ASA+**	solvent accessible molecular surface area due to atoms with positive parial charge [11]
14	ASA-	solvent accessible molecular surface area due to atoms with negative parial charge [11]
15	ASA_H	solvent accessible molecular surface area due to atoms with hydrophobic properties [11]
**16**	**ASA_P**	solvent accessible molecular surface area due to atoms with polar properties [11]
**17**	**FASA+**	fractional solvent accessible molecular surface area calculated as ASA+/ASA [11]
**18**	**FASA-**	fractional solvent accessible molecular surface area calculated as ASA-/ASA [11]
19	FASA_H	fractional solvent accessible molecular surface area calculated as ASA_H/ASA [11]
20	FASA_P	fractional solvent accessible molecular surface area calculated as ASA_P/ASA [11]
21	FCASA+	Positive charge weighted surface area, ASA+ * maximum positive partial charge [11]
22	FCASA-	Negative charge weighted surface area, ASA- * maximum negative partial charge [11]
23	glob	globularity [11]
**24**	**std_dim1**	largest standardized dimension [11]
**25**	**std_dim2**	second largest standardized dimension [11]
26	std_dim3	third largest standardized dimension [11]
**27**	**logS**	log of calculated water solubility [11]
**28**	**E_sol**	solvation Energy [11]
29	logP(o/w)	log of calculated octanol/water partition coefficient [11]
30	SlogP	log of calculated octanol/water partition coefficient [11]
31	ASAN1	fractional accessible surface area due to atoms in partial charge interval 0 to -0.05 e [7]
32	ASAN2	fractional accessible surface area due to atoms in partial charge interval -0.05 to -0.1 e [7]
33	ASAN3	fractional accessible surface area due to atoms in partial charge interval -0.1 to -0.15 e [7]
34	ASAN4	fractional accessible surface area due to atoms in partial charge interval -0.15 to -0.2 e [7]
**35**	**ASAN5**	fractional accessible surface area due to atoms in partial charge interval -0.2 to -0.25 e [7]
**36**	**ASAN6**	fractional accessible surface area due to atoms in partial charge interval -0.25 to -0.3 e [7]
37	ASAN7	fractional accessible surface area due to atoms in partial charge interval <-0.30 e [7]
**38**	**ASAP1**	fractional accessible surface area due to atoms in partial charge interval 0 to 0.05 e [7]
39	ASAP2	fractional accessible surface area due to atoms in partial charge interval 0.05 to 0.1 e [7]
**40**	**ASAP3**	fractional accessible surface area due to atoms in partial charge interval 0.1 to 0.15 e [7]
**41**	**ASAP4**	fractional accessible surface area due to atoms in partial charge interval 0.15 to 0.2 e [7]
**42**	**ASAP5**	fractional accessible surface area due to atoms in partial charge interval 0.2 to 0.25 e [7]
43	ASAP6	fractional accessible surface area due to atoms in partial charge interval 0.25 to 0.3 e [7]
44	ASAP7	fractional accessible surface area due to atoms in partial charge interval >0.3 e [7]

## References

[1] World Health Organization, 2004 Global burden of disease report update, 2004.

[2] World Health Organization, 2007. http://www.who.int/features/factfiles/neglected_tropical_diseases/en/index.html..

[3] Hoet S., Opperdoes F., Brun R., Quetin-Leclercq J. (2004). Natural products active against African trypanosomes: a step towards new drugs. Nat. Prod. Rep..

[4] Schmidt T.J., Willuhn G., Brun R., Khalid S.A. (2002). Antitrypanosomal activity of Helenalin and some related Sesquiterpene Lactones. Planta Med..

[5] Nour A.M.M., Khalid S.A., Kaiser M., Brun R., Abdallah W. E., Schmidt T.J. (2009). The antiprotozoal activity of sixteen Asteraceae species native to Sudan and bioactivity-guided isolation of xanthanolides from *Xanthium brasilicum* Vell. Planta Med..

[6] Schmidt T.J. (1999). Quantitative structure-cytotoxicity relationships within a series of helenanolide type sesquiterpene lactones. (Helenanolide Type Sesquiterpene Lactones, IV.). Pharm. Pharmacol. Lett..

[7] Schmidt T.J., Heilmann J. (2002). Quantitative Structure-Cytotoxicity Relationships of Sesquiterpene Lactones derived from Partial Charge (Q)-based Fractional Accessible Surface Area Descriptors (Q_frASAs). Quant. Struct.-Act. Relat. (QSAR).

[8] Schmidt T.J. (1999). Toxic Activities of Sesquiterpene Lactones – Structural and Biochemical Aspects. Current Org. Chem..

[9] Schmidt T.J., Atta-ur-Rahman (2006). Structure-Activity Relationships of Sesquiterpene Lactones. Studies in Natural Products Chemistry.

[10] Beekman A.C., Woerdenbag H.J., van Uden W., Pras N., Konings A.W.T., Wikström H., Schmidt T.J. (1997). Structure-Cytotoxicity Relationships of some Helenanolide-Type Sesquiterpene Lactones. J. Nat. Prod..

[B11-molecules-14-02062] 11.*MOE - Molecular Operating Environment v. 2008.10*, available from Chemical Computing Group, Montreal, Canada: http://www.chemcomp.com/; For details on molecular descriptors see: http://www.chemcomp.com/journal/descr.htm

[12] The Unscrambler, v. 9.2.

[13] Fleming I. (1976). Frontier Orbitals and Organic Chemical Reactions.

[14] De Carvalho P.B., Ramos D.C.C., Cotrim P.C., Ferreira E.I. (2003). Synthesis and in vitro evaluation of potential anti-leishmanial targeted drugs of pyrimethamine. J. Pharm. Sci..

[15] Chung M.-C., Carvalho Guido R.V., Martinelli T.F., Ferreira Goncalves M., Carneiro Polli M., Alves Botelho K.C., Aparecida Varanda E., Colli W., Mirando M.T.M., Ferriera E.I. (2003). Synthesis and in vitro evaluation of potential antichagasic hydroxymethylnitrofurazone (NFOH-121): a new nitrofurazone prodrug. Bioorg. Med. Chem..

[16] Willuhn G., Merfort I., Paßreiter C.M., Schmidt T.J., Hind D.J.N., Jeffrey C., Pope G.V. (1994). Chemistry and Systematics of the Genus Arnica. Advances in Compositae Systematics.

[17] Budesinsky M., Saman S. (1995). Carbon-13 NMR Spectra of Sesquiterpene Lactones. Ann. Rep. NMR Spect..

[18] Bohlmann F., Mahanta P.K., Jakupovic J., Rastogi R.C., Natu A.A. (1978). New Sesquiterpene Lactones from *Inula* species. Phytochemistry.

[19] Matile H., Pink J.R.L., Lefkovits I., Pernis B. (1990). *Plasmodium falciparum* malaria parasite cultures and their use in immunology. Immunological Methods.

[20] Räz B., Iten M., Grether-Bühler Y., Kaminsky R., Brun R. (1997). The Alamar Blue assay to determine drug sensitivitiy to African Trypanosomes (*T. b. rhodesiense* and *T. b. gambiense*) in vitro. Acta Trop..

[21] Buckner F.S., Verlinde C.L., La Flamme A.C., Van Voorhis W.C. (1996). Efficient technique for screening drugs for activity against *Trypanosoma cruzi* using parasites expressing beta-galactosidase. Antimicrob. Agents Chemother..

[22] Cunningham I. (1977). New culture medium for maintenance of tsetse tissues and growth of trypanosomatids. J. Protozool..

